# Malpractice and Medical Testimony

**Published:** 1876-10

**Authors:** 


### Editorial.

### Malpractice and Medical Testimony.

A recent suit for malpractice in this city has attracted so much attention in medical circles, that we give in our miscellaneous department a synopsis of the medical testimony, condensed from the notes of the court stenographer.

The decision of the case turned upon the question of whether there had been a dislocation of the hip as claimed by the plaintiff. The defense claimed that the injury was not a dislocation, but a fracture, and that it was of such character as to escape detection. The defendant's counsel asked that four experts be appointed by the court to examine the plaintiff and give testimony as to his present condition. This request was acceded to and Drs. E. M. Moore, of Rochester, James P. White, Thomas F. Rochester, and Julius F. Miner, of Buffalo, appointed. The result of their examination is given in the testimony published elsewhere. Dr. Moore was prevented from testifying by a severe family affliction, but we understand he fully agreed with his fellow appointees.

We are very sorry to say that the chief witness on the part of the plaintiff has attempted to make himself and the public believe that the testimony of the experts appointed by the court was chiefly given with a view of destroying his reputation, and taking away from him the credit of having reduced a dislocation of the head of the femur of nine months' duration. As his own testimony shows that he did not reduce the dislocation, we do not see how he can consistently make this plea. It is certainly a very serious charge for him to say that four men of large experience and extensive reputation would deliberately perjure themselves to detract from him a certain amount of merit, which after all, by his own showing, does not belong to him. For Dr. Gay personally, we have the kindest feelings, and our readers have formed a certain acquaintance with him through our columns. We are, therefore, very sorry that he should take this position. Medical men frequently differ as to matters of diagnosis and treatment, but they can and do have these differences without any personal feeling in the matter. If Dr. Gay honestly thinks there was, on the twenty-sixth of May last, a dislocation, we respect his opinion, but we also think that Drs. White, Miner and Rochester, as well as the other medical witnesses in the case on behalf of the defendant, are equally honest in saying that the injury with which Bergman is now suffering, and has been probably since the receipt of the injury in August, 1875, is a fracture of the neck of the femur.

Dr. Gay now candidly says that he did not reduce the dislocation, but that the head of the femur is in the ischiatic notch. He has, as will be seen by reference to the Proceedings of the Buffalo Medical Association, promised a full report of the case in the future, and as a brief reference to it as a successful reduction is found in the Proceedings of the American Medical Association which might lead to erroneous conclusions, we hope he will do so. One lesson which all can learn from it, is, the inadvisability of reporting cases until the diagnosis and results of treatment are fully established. We suspect, if this rule were adhered to, the statistics of many surgical operations, as well as of the termination of certain cases of disease, would be materially changed.

